# Participant Characteristics as Moderators of the Effects of Cognitive Behavioral Interventions on Concerns About Falling: Secondary Analyses of Two Randomized Controlled Trials

**DOI:** 10.1177/07334648231165904

**Published:** 2023-04-06

**Authors:** Marlot Kruisbrink, G.A. Rixt Zijlstra, Rik Crutzen, Tanja A. C. Dorresteijn, Bjorn Winkens, Gertrudis I. J. M. Kempen

**Affiliations:** 1 Department of Health Services Research, Care and Public Health Research Institute (CAPHRI), Faculty of Health, Medicine and Life Sciences, 5211 Maastricht University, Maastricht, the Netherlands; 2 Department of Health Policy & Research, Public Health Service Flevoland (GGD Flevoland), Lelystad, the Netherlands; 3 Department of Health Promotion, Care and Public Health Research Institute (CAPHRI), Faculty of Health, Medicine and Life Sciences, 5211 Maastricht University, Maastricht, the Netherlands; 4 Department of Strategy and Policy, Public Health Service Limburg North, Venlo, the Netherlands; 5 Department of Methodology and Statistics, Care and Public Health Research Institute (CAPHRI), Faculty of Health, Medicine and Life Sciences, 5211 Maastricht University, Maastricht, the Netherlands

**Keywords:** Falls, intervention, quantitative methods, concerns about falling, moderation

## Abstract

Effects of interventions may vary among participants. We explored whether participant characteristics were moderators of the effects of two cognitive behavioral interventions on concerns about falling (CaF) in older community-dwelling people. Secondary analyses of two RCTs were performed, concerning the group intervention A Matter of Balance - Netherlands (AMB-NL, *n* = 540) and individual AMB - Home (*n* = 389) intervention. Marginal models were used to assess moderation. Analyses included single moderator and multiple moderator models containing multiple moderators at once. A total of 19 characteristics were assessed. Moderating effects were found for living situation, fall history, symptoms of depression, perceived general health, ADL disability, cognitive status, and consequences of falling—loss of independence subscale. Effects varied by intervention, time point, and type of model.

**What this paper adds**

• The results of this study add to the limited evidence on participant characteristics that moderate the effects of cognitive behavioral interventions on concerns about falling in older community-dwelling people.
• The effects of the cognitive behavioral group intervention A Matter of Balance - Netherlands (AMB-NL) are moderated by symptoms of depression, cognitive status, ADL disability, consequences of falling—loss of independence subscale, and perceived general health.
• Perceived general health, fall history, and living situation moderated the effects of the individual cognitive behavioral intervention A Matter of Balance - Home (AMB-Home).

**Applications of study findings**

• The results demonstrate the importance of including people with disabilities in recruitment efforts.
• Results give clues for potential improvements to the interventions. For example, in AMB-home, more intervention-specific social support may be useful for those that live alone.

## Introduction

Among older people, concerns about falling (CaF)—also called fear of falling—is common. Prevalence typically ranges between 21% and 85% (Scheffer et al., 2008). CaF is associated with poor physical functioning, activity avoidance, low social participation, and lower quality of life. It may also present a problem for independence (Cumming et al., 2000; Howland et al., 1998; Meulen et al., 2014; Scheffer et al., 2008; Schoene et al., 2019). Interventions based on cognitive behavioral principles such as challenging maladaptive thoughts or graded exposure can reduce CaF. A meta-analysis of 15 studies evaluating cognitive behavioral interventions (Chua et al., 2019) showed short- and long-term effects but small or moderate reductions in CaF, thus leaving room for improvement.

Participant characteristics are important to consider for intervention optimization as some people benefit more from interventions than others. For example, a study evaluating an exercise program showed a larger reduction in participants’ fear of falling when they received less social support at baseline (Fukukawa et al., 2008). Potentially, an exercise program may be particularly suitable for those that impose self-induced exercise restrictions due to lack of social support. Another example is a study by Tennstedt and colleagues (2001), which concerned a cognitive behavioral group intervention involving restructuring misconceptions, goal setting, environmental changes, and physical activity. Compliers who had higher baseline fear of falling scores, higher levels of physical and social functioning, and higher perceived control over falling benefited more from the intervention. Thus, the intervention was most beneficial for those with greater room for improvement, whose participation was not hindered by dysfunction, and who believed they could do something about falling. Such findings can facilitate the screening of participants for interventions. By targeting certain groups, intervention effects can be optimized. Alternatively, interventions can be modified to accommodate groups that benefit less.

Little is known about participant characteristics that moderate the effects of cognitive behavioral interventions on CaF in older community-dwelling people. The objective of this study was to explore whether participant characteristics are moderators of the effects of cognitive behavioral interventions on CaF in older community-dwelling people.

## Materials and Methods

This study concerns a secondary data-analysis of two randomized controlled trials (RCTs) of cognitive behavioral interventions. This study was pre-registered at Open Science Framework (OSF; https://osf.io/vxy6u). Our main focus was the group intervention A Matter of Balance - Netherlands (AMB-NL, Trial ID: ISRCTN43792817, and ethical review: MEC 07-3-064). We also analyzed the data of the more recent AMB-Home intervention (Trial ID: NCT01358032 and ethical review: METC 01-075). AMB was developed in the United States of America (Tennstedt et al., 1998), as a cognitive behavioral group intervention to reduce CaF and associated activity restriction. AMB has been adapted to the Dutch setting in AMB-NL (Zijlstra et al., 2006). AMB-Home is the home-based version of AMB-NL (Dorresteijn et al., 2011), developed to accommodate people with health problems, and/or a preference for an individual approach or an intervention at home (Dorresteijn et al., 2012). AMB-NL and AMB-Home have demonstrated effectiveness on CaF and are currently applied in the Netherlands (Trimbos Instituut, n. d.). Therefore, insight into participant characteristics as moderators is of importance to current practice.

A short description of both trials is given below. More information can be found elsewhere (Dorresteijn et al., 2011; Zijlstra et al., 2005). Participants provided written informed consent (Dorresteijn et al., 2011; Zijlstra et al., 2005).

### Participants and Procedures

In both RCTs, random samples of potential participants were obtained via municipality registers. In AMB-NL, community-dwelling older people (≥70 years) were eligible to participate if they had some CaF and activity avoidance and lived in the south of the Netherlands. Exclusion criteria were being confined to bed, being wheelchair dependent, participating in other intervention studies**,** or waiting for nursing home admission. Criteria for AMB-Home were similar, but participants also had to perceive their health as fair or poor. Furthermore, they could not have substantial cognitive, hearing, or visual impairment. In both trials, participants were assigned to an intervention group or usual care group. Randomization was performed by an independent researcher (AMB-NL) or external agency (AMB-Home). Participants and facilitators were not blinded to group assignment.

In AMB-NL, 280 people were allocated to the intervention and 260 to the control group, 169 (60.4%) and 209 (80.4%) completed the trial, respectively. In AMB-Home, 194 people were allocated to the intervention and 195 people to the control group, of which 133 (68.6%) and 162 (83.1%) completed the trial, respectively. Baseline characteristics are shown in supplementary material Table 1. The samples were predominantly female and on average 78 years. The majority lived alone. Comparing the samples, AMB-NL had relatively more people with impaired hearing or vision. AMB-Home had no people who rated their health as good and had more people with a chronic condition. These differences reflect the inclusion criteria.

### Interventions

AMB-NL is an 8 week intervention with weekly 2 hr group sessions (Zijlstra et al., 2005). The main topics include an introduction to CaF and the program, thoughts and their influence on CaF, physical exercise, assertiveness, managing concerns through physical exercise and cognitive restructuring, fall-risk behaviors, fall hazards in the home and community, and safe behavior. Four main strategies are used: restructuring misconceptions, goal setting, changing the home environment, and physical exercise. Participants reflect on their own situation and discuss their own perspectives. The group format also allows for feedback, social support, and comparison. Furthermore, there is attention for the implementation of strategies into daily life. AMB-NL includes a booster session 6 months after the last session.

AMB-Home consists of seven sessions. The first four take place weekly and the last three sessions every 2 weeks. There are three home visits and four telephone contacts (Dorresteijn et al., 2013). The topics and strategies are similar to those of AMB-NL, but the physical exercises were replaced by an activity under the supervision of a nurse (exposure) and motivational interviewing was incorporated. Although social support and comparison are less pronounced than in the group intervention, participants in AMB-Home could invite a significant other to be present at the home visits for support and motivation between sessions (Dorresteijn et al., 2011).

Participants in the control groups received care as usual. It is likely these participants received no intervention (Dorresteijn et al., 2016; Zijlstra et al., 2009).

### Rationale for Chosen Moderators

Little is known about participant characteristics that moderate the effects of cognitive behavioral interventions on CaF in older community-dwelling people. The previously mentioned study by Tennstedt and colleagues (2001) is one of the few studies on this subject. Yet, in addition to baseline CaF, physical and social functioning, and perceived control over falling, identified by Tennstedt et al. (2001), other characteristics are of interest. First, decreased cognitive functioning, hearing and visual problems, lack of mastery, and lack of social support occur more often among older people (Ellis, 1999; Evans, 2007). Second, following the theory of planned behavior, several socio-cognitive variables such as attitudes and perceived norms may influence the intention to engage in the intervention (Ajzen, 1991). Third, considering other populations and outcomes such as depression and anxiety, research shows that sex, age, educational level, living status, and comorbidity may influence outcomes of cognitive behavioral interventions (Beltman et al., 2010; Button et al., 2015; Gitlin et al., 2008; Hoifodt et al., 2015; Keeley et al., 2008; Knopp et al., 2013; Maher et al., 2010; Porter & Chambless, 2015; Raffin et al., 2009; Springer et al., 2018; Wetherell et al., 2005). Lastly, variables strongly associated with CaF may be moderators, such as fall history and perceived general health (Denkinger et al., 2015).

### Measurements

Data were collected with self-report questionnaires and telephone interviews. Outcome assessors were trained and blinded to group assignment. Measurements were performed during screening, at baseline and directly after the intervention (T1). The AMB-NL trial had a follow-up measurement at 6 (T2) and 12 months after the intervention (T3). The AMB-Home trial had a follow-up measurement 7 months after the intervention (T2).

### Outcome Measures

Most outcome measures of the trials were identical; only not identical outcome measures are described separately below. The most favorable scores are underlined.

#### Dependent Variable

CaF was measured by asking participants how concerned they are while carrying out activities of daily living (1 = not at all concerned, 4 = very concerned). An adapted version of the 10-item Falls Efficacy Scale (FES) questionnaire, with four additional outdoor activity items, was used in AMB-NL (Zijlstra et al., 2005). In AMB-Home, CaF was measured with the 16-item Falls Efficacy Scale-International (FES-I; (Yardley et al., 2005)). A sum score was calculated, resulting in a range of 14 to 56 for the adapted FES and a range of 16 to 64 for the FES-I.

#### Moderators

##### Demographics

Demographic variables were assessed by questionnaire during eligibility screening: age, sex (male/female), living situation (categorized as living alone/not living alone), and educational level (categorized as low/middle/high).

##### Health Status

One item of the MOS Short-Form General Health Survey was used to assess perceived general health (categorized as good/fair/poor). Fall history in the past 6 months was assessed with one item (categorized as never/once/more than once). Disabilities in activities of daily living (ADL) were measured with the ADL subscale of the Groningen Activity Restriction Scale (GARS) (11 items, sum score 11–44). Chronic medical conditions were assessed with a 5-item questionnaire (categorized as at least one chronic condition/no chronic conditions). Cognitive status was assessed with the 25-item Telephone Interview for Cognitive Status (TICS) in AMB-NL (sum score 0–41). Impaired vison was assessed with a 2-item questionnaire in AMB-NL and a 1-item questionnaire in AMB-Home (categorized as impaired/not impaired). Impaired hearing was assessed similarly. Symptoms of depression and feelings of anxiety were measured with the two subscales of the Hospital Anxiety and Depression scale (each subscale contains seven items, sum score 0–21).

##### Socio-Cognitive

Mastery was assessed with the 7-item personal mastery scale (sum score 7–35). Social support was measured with the 12-item Social Support List of Interactions (SSL12-I) (sum score 12–48). Perceived control over falling was assessed with the 4-item Perceived Control over Falling (PCOF) scale (sum score 4–20). Perceived consequences of falling were measured with the Consequences of Falling (CoF) scale measuring the loss of functional independence and damage to identity (each subscale contains six items, sum score 6–24).

### Analysis

We used marginal models for repeated measures with restricted maximum likelihood (REML) estimation and an unstructured covariance structure for the repeated measures to assess whether a variable moderated the effect of the intervention on CaF. Syntaxes are available on OSF (https://doi.org/10.17605/OSF.IO/ENZB6). Marginal models were applied to the data of each RCT separately.

Missing values were handled according to the administration rules of each measure (e.g., mean imputation was performed at the level of the scale when the number of missing items did not exceed the maximum number of missing items following the administration rules). If no guidelines were available, a maximum of 25% missing values was used for AMB-Home as a rule of thumb. For AMB-NL, a maximum of 15% missing values was used, except for the adapted FES, for which 25% of missing values was allowed. For both datasets, a likelihood-based approach, assuming missingness at random, was used for remaining missing values in the outcome variables. A significance level of .05 was used to determine whether moderation was significant. No multiple testing correction was performed; we considered these analyses to be exploratory. Analyses were performed with IBM SPSS Statistics for Windows version 25 (Armonk, NY, USA; IBM Corp). The fixed parts of the single moderator and multiple moderator models are described below. A random intercept on participant level was not specified. Because an unstructured covariance structure for the repeated measures was used, this would be redundant.

#### Single Moderator Models

In the single moderator models, one moderator was entered at a time. The models consisted of the moderator at baseline, the group assignment variable, and the categorical ‘time’ variable (using dummy variables; AMB-NL: three measurements, AMB-Home: two measurements). Analyses were corrected for baseline CaF and community. The following interactions were included as well: group x time, moderator x time, group x moderator, and group x time x moderator. We analyzed the following potential moderators measured at baseline: CaF, age, sex, living situation, educational level, ADL disability, chronic medical conditions, cognitive status (only for AMB-NL), visual impairment, hearing impairment, symptoms of depression, feelings of anxiety, mastery, social support, perceived control over falling, and perceived consequences of falling (both subscales). In the OSF pre-registration, fall history and perceived general health were not included as moderators. There is little evidence on these two variables as moderators of cognitive behavioral interventions, but we added them to the analyses due to their association with CaF.

#### Multiple Moderator Model

In these exploratory analyses, we wanted to check whether moderation effects would remain significant when multiple moderators were included in the model. Therefore, in addition to models with one moderator, more elaborate models with multiple moderators were specified as well. Like the single moderator models, the multiple moderator model included moderators at baseline, group, time, CaF at baseline, community, and three- and two-way interactions between group, time, and moderator. However, the multiple moderator model included multiple moderators and interactions with moderators at once. We included moderators that showed promising results in the single moderator models, which was defined as a *p*-value ≤.10 for the two-way interaction group x moderator or the three-way interaction group x moderator x time.

The multiple moderator model included the additional variables age, sex, fall history, perceived general health, educational level, and living situation. The Supplementary Material has a list of all included variables.

#### Sensitivity Analyses

Attending five sessions has previously been considered as compliant (Dorresteijn et al., 2016). Intention-to-treat analyses were performed. However, some variables may sort their effects through intention, motivation, and compliance. Therefore, we performed separate analyses in the group that attended five or more sessions (“on-treatment” analyses).

## Results

### A Matter of Balance - Netherlands

Most variables were not significant moderators. Symptoms of depression, cognitive status, ADL disability, consequences of falling (loss of independence subscale), and perceived health showed significant moderating effects varying by time point and type of analysis. In general, the single moderator models showed more significant moderators than the multiple moderator model. Additionally, the on-treatment analyses showed more moderators than the intention-to-treat analyses. Table 1 presents an overview. Details are described below.

**Table 1.** Overview of significant moderators in A Matter of Balance - Netherlands and A Matter of Balance - Home.

	Single Moderator Models			Multiple Moderator Model		
	T1	T2	T3	T1	T2	T3
A Matter of Balance - Netherlands						
Intention-to-treat	• Symptoms of depression	• Cognitive status				
On-treatment	• Symptoms of depression	• Cognitive status	• Cognitive status		• Symptoms of depression	
	• ADL disability	• Symptoms of depression				
	• Consequences of falling^a^					
	• Perceived general health					
A Matter of Balance - Home						
Intention-to-treat	• Perceived general health	• Fall history				
On-treatment	• Perceived general health	• Fall history		• Living situation		
	• Living situation					

^a^Loss of independence subscale.

#### Single Moderator Models, Intention-to-Treat Outcomes

**Figure 1.**
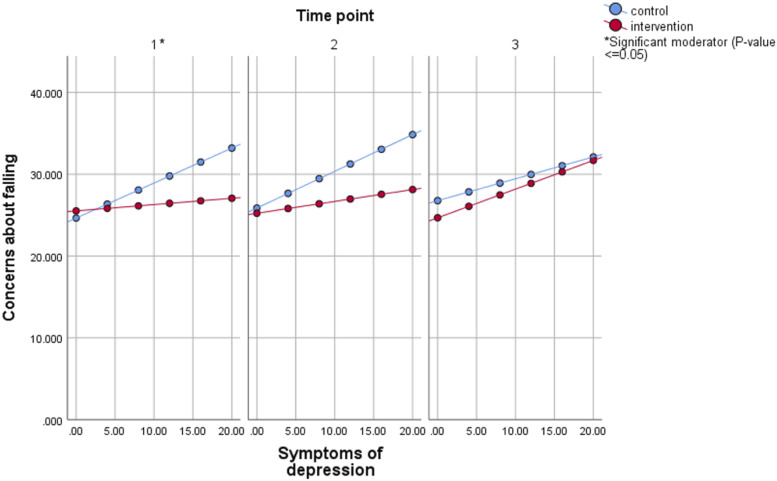
Estimated mean concerns about falling for different values of symptoms of depression in the single moderator model, intention-to-treat analysis of A Matter of Balance - Netherlands. Model included group, time, community, baseline concerns, depression, group*time, depression*time, group*depression, and group*time*depression. Sum scores range from 14 to 56 for concerns about falling and 0 to 21 for symptoms of depression (the underlined score is the most favorable score).

**Figure 2.**
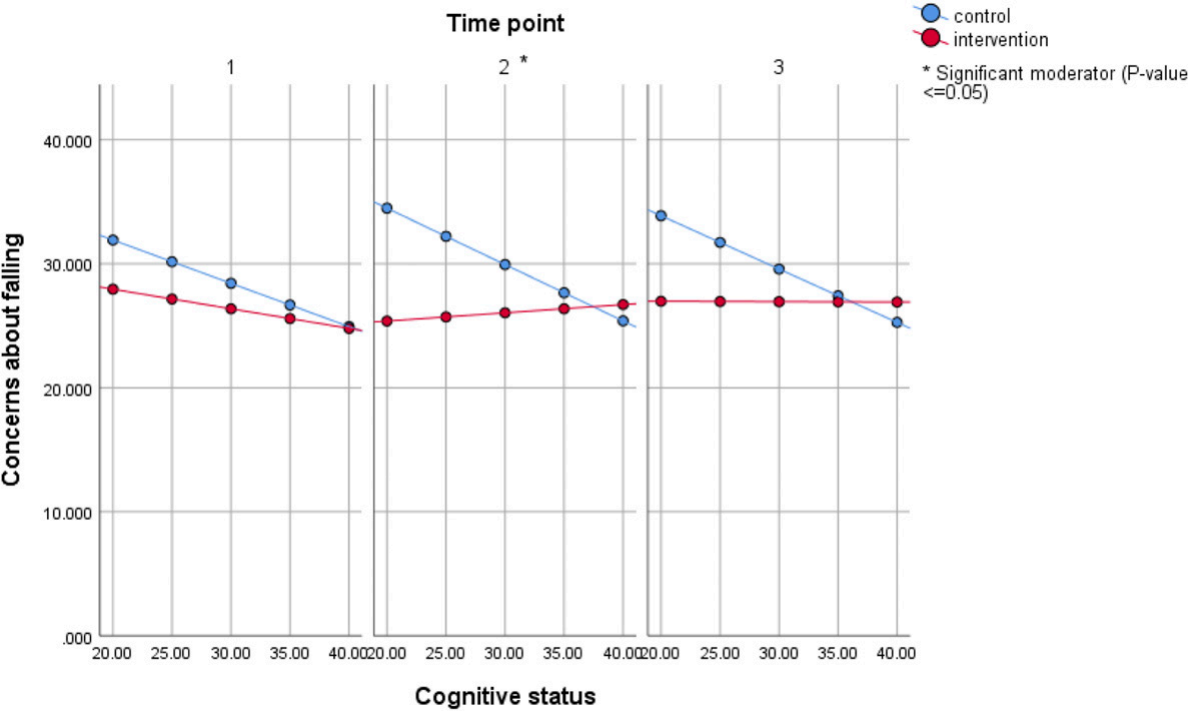
Estimated mean concerns about falling for different values of cognitive status in the single moderator model, intention-to-treat analysis of A Matter of Balance - Netherlands. Model included group, time, community, baseline concerns, cognition, group*time, cognition*time, group*cognition, and group*time*cognition. Sum scores range from 14 to 56 for concerns about falling and 0 to 41 for cognitive status (the underlined score is the most favorable score).

Symptoms of depression was a significant moderator at T1 in the single moderator model. CaF was similar in the intervention and control group for lower levels of depression symptoms (Figure 1). As symptoms of depression increased, so did CaF, but this was more pronounced in controls. This effect was also visible at T2, but it was not significant. Furthermore, the intervention and control group had similar levels of CaF for high levels of cognitive status at T2 (Figure 2). However, as cognitive status decreased, controls showed a higher level of CaF than those in the intervention group. Results were similar for T3, but the moderating effect was not significant.

#### Single Moderator Models, on-Treatment Outcomes

Moderating effects of symptoms of depression and cognitive status were similar in the on-treatment analyses, but effects were significant at more time points (supplementary material Figure 1). Effects were also more pronounced for symptoms of depression; as symptoms of depression increased, the intervention group showed a decrease in CaF.

Furthermore, compared to the intervention group, CaF increased more in the control group for increasing levels of ADL disability and perceived consequences of falling (loss of independence subscale) at T1 (supplementary material Figure 1). The intervention effect also differed between categories of perceived general health at T1. There was a significant intervention effect for those in fair health: the adjusted mean CaF was significantly lower in the intervention group than in the control group (supplementary material Table 2, mean difference [95%CI]: −3.68 [−5.46; −1.91]). Such an effect was not found for those in good health.

#### Multiple Moderator Model, Intention-to-Treat Outcomes

There were no significant moderating effects in the intention-to-treat analyses of the multiple moderator model.

#### Multiple Moderator Model, on-Treatment Outcomes

Depression symptom was a significant moderator in the on-treatment analysis of the multiple moderator model, at T2 only (supplementary material Figure 2).

### AMB-Home

Similar to AMB-NL, in AMB-Home most variables were not significant moderators, and there were more significant moderators in the single moderator models and on-treatment analyses (Table 1). Perceived general health, fall history, and living situation showed significant moderating effects varying by time point and type of analysis.

#### Single Moderator Models, Intention-to-Treat Outcomes

At T1, a significant intervention effect is found among those in fair health (Table 2, mean difference [95%CI]: −4.24 [−5.90; −2.59]) but not those in poor health. Additionally, a significant effect is shown in those who have never fallen or fallen more than once at T2 Table 2, mean difference never category [95% CI]: −2.86 [−5.67; −0.05]; mean difference more than once category [95% CI]: −6.75 [−9.78; −3.71]). In contrast, the analyses showed no significant effect in those who have fallen once. Similar findings are shown for T1, without being significant.

**Table 2.** Intervention effects of A Matter of Balance - Home in categories of significant moderators. Results are from the intention-to-treat analysis.

Moderator	Time point^a^	Categories	Single Moderator Model	Multiple Moderator Model
			Adjusted Mean Difference (95%CI)^b^	Adjusted Mean Difference (95%CI)^c^
Perceived general health	1^d^	Fair	−4.24 (−5.90; −2.59)^e^	−3.18 (−6.08; −0.27)^e^
		Poor	1.02 (−3.89; 5.92)	0.90 (−4.38; 6.17)
	2	Fair	−4.52 (−6.35; −2.68)^e^	−5.49 (−8.83; −2.16)^e^
		Poor	−0.11 (−5.52; 5.30)	−0.67 (−6.48; 5.14)
Falls in the past 6 months	1	Never	−5.30 (−7.82; −2.79)^e^	−3.42 (−7.53; 0.70)
		Once	−2.08 (−5.07; 0.92)	−0.01 (−4.11; 4.08)
		More than once	−2.85 (−5.58; −0.12)^e^	0.01 (−3.97; 3.99)
	2^d^	Never	−2.86 (−5.67; −0.05)^e^	−2.70 (−7.29; 1.89)
		Once	−2.15 (−5.52; 1.23)	−1.38 (−5.98; 3.23)
		More than once	−6.75 (−9.78; −3.71)^e^	−5.17 (−9.55; −0.78)^e^

^a^Time point 1 = directly after the intervention, 2 = 7 months after the intervention.

^b^Mean difference = intervention–control. Single moderator model, adjustments for group, time, community, baseline concerns, moderator, group*time, moderator*time, group*moderator, and group*time*moderator.

^c^Mean difference = intervention–control. Multiple moderator model, for adjustments please see the main text of the article.

^d^There is a significant difference (*p*-value ≤.05) in intervention effects between categories of the moderator in the single moderator model.

^e^There is a significant adjusted mean difference between the intervention and control group.

#### Single Moderator Models, On-Treatment Outcomes

Perceived health and fall history were still significant moderators in the on-treatment analyses (supplementary material Table 3). Additionally, effects in categories of living situation differed significantly at T1 (mean difference alone category [95% CI]: −2.33 [−4.50; −0.15]; mean difference not alone category [95% CI]: −5.80 [−8.44; −3.16]).

#### Multiple Moderator Model, Intention-to-Treat Outcomes

None of the variables significantly moderated effects in the intention-to-treat analysis of the multiple moderator model (Table 1).

#### Multiple Moderator Model, On-Treatment Outcomes

Living situation was a significant moderator at T1 (supplementary material Table 3, mean difference alone category [95%CI]: .84 [−2.97; 4.65]; mean difference not alone category [95%CI]: −3.92 [−7.92; 0.09]).

## Discussion

We explored moderators of the effects of AMB-NL and AMB-Home, two cognitive behavioral interventions for managing CaF. Many participant characteristics were not significant moderators in any model. None of the characteristics in the multiple moderator models were significant on the long term. However, a few participant characteristics showed significant moderating effects. Moderating effects depended on the specific analysis, that is, on the intervention, time point, and type of analysis (intention-to-treat or on-treatment).

### Demographic

In both the single moderator and multiple moderator models, the effect of the AMB-Home intervention was larger for those living with someone else. Qualitative research on cognitive behavioral therapy (CBT) for depression has demonstrated that cohabitating can be a source of motivation, which can impact compliance (Wilhelmsen et al., 2013). Alternatively, having another person nearby may have helped to engage better with the intervention content. Something similar was found in a qualitative study on CBT for insomnia (Dyrberg et al., 2021), in which family members helped to implement instructions from therapists. Fall history was also a significant moderator. People who fall more often may not perform activities in a safe way (Butler et al., 2014; Zecevic et al., 2009). AMB specifically gives attention to safe behavior and thus these people may benefit from the intervention. There were slightly more fallers in AMB-Home than in AMB-NL, which may explain why this was only found for AMB-Home.

### Health Status

Of the evaluated health variables, symptoms of depression, perceived health, ADL disability, and cognitive status were moderators.

In AMB-NL, symptoms of depression was a moderator. CaF barely increased with increasing levels of depression symptoms. When we only consider compliers, the intervention was even more effective for those with more severe depression symptoms at baseline. The mechanism behind this is uncertain. Previous studies indicate that depressive symptoms and CaF are associated, although it is still uncertain how exactly they interact (Gambaro et al., 2022). The result of the current study could imply that the skills taught in AMB-NL—such as cognitive restructuring, problem solving and exposure—were transferred to other situations in the older person’s life, resulting in decreased depressive symptoms and an enhanced intervention effect on CaF.

Perceived health was a moderator in both interventions. For AMB-NL, CaF was effectively reduced in those in fair health but not those in good health. People in fair health may be less confident (Lach, 2005) and may have misconceptions about activities they can safely perform. Disparities between perceived and physiological fall risk can occur in older people (Delbaere et al., 2010). The intervention addresses restructuring misconceptions and safely performing activities, and people in fair health could benefit more. Additionally, AMB-NL is a group intervention, and social comparison may motivate participants to try activities. Previous research has demonstrated that social modelling may motivate older people to perform physical activity (Booth et al., 2000; Warner et al., 2011). A similar social modelling mechanism could explain the moderating effect of ADL-disability for AMB-NL. In contrast, poor health may limit possibilities to effectively engage in the intervention; AMB-Home was effective in those in fair health but not those in poor health.

Lastly, for AMB-NL, CaF did not vary in the intervention group much for different levels of cognitive status. In contrast, in the control group, those with a poor cognitive status had higher levels of CaF. AMB-NL contains cognitively stimulating elements. As cognition and CaF appear associated (Noh et al., 2019; Uemura et al., 2015), improvements in cognition may lead to improvements in CaF. In the current study, we did not take into account changes in cognitive status over time; if those with poor cognitive status improved due to the intervention, this may explain why CaF in the intervention group was not higher.

### Socio-Cognitive

One of the socio-cognitive variables was a significant moderator. In the control group of the AMB-NL trial, CaF increased with increasing scores of the consequences of falling—loss of independence subscale. This was not the case in the intervention group. The intervention participants that scored high on the consequences of falling scale at baseline may have adopted more realistic thoughts during the intervention if they learned that falls do not necessarily lead to negative outcomes and that they can reduce their risk of falling.

### Strengths and Limitations

The RCTs included relatively large samples, and we explored many potential moderators from the demographic, health, and socio-cognitive domains. Two types of models were used, with limited and elaborate adjustments. However, this study was also subject to limitations. Because we used existing data, we were limited to the characteristics that have been measured in the AMB-NL and AMB-Home trials and no sample size calculations were performed. Additionally, in these exploratory analyses, we used a significance limit of 0.05 to test interaction effects and did not use a multiple testing correction. Therefore, there is a risk of type I and type II errors.

### Implications

In some of the analyses, more ADL disability, more symptoms of depression, and low cognitive status at baseline were associated with more CaF in the control group, while CaF in the intervention group increased less strongly. Although the underlying mechanisms for these results warrant more investigation, the recruitment materials for the current interventions may be adapted in order to provide guidance on recruiting participants with these characteristics, such as using multimodal forms of communication (not only written information), placing brochures and posters in waiting rooms of health care providers, and collaborating with agencies for independent living to directly contact older people (Banas et al., 2019). Although recruiting people with these characteristics may be challenging, participation may be advantageous for them and it is important to make efforts to include them. Other moderators, such as sex, living situation, and fall history, are not easily modifiable, but may be highlighted in the manuals with a textbox. For example, for those with a fall history, it may prove more beneficial to focus on the intervention components safe behavior and increasing physical activity, or for those that live alone, more intervention specific social support may be needed.

### Further Research

Studies often report average effects, but older people are a highly heterogeneous group and effects may differ according to participant characteristics. In addition to the characteristics examined here, other potential moderators warrant exploring, such as participant’s expectations on intervention benefits prior to the start of the intervention. For group interventions, the composition and characteristics of the group may be considered (Wilson et al., 2019). Secondary data-analysis can be a suitable method for future research into potential moderators. Lastly, AMB-NL and AMB-Home are similar in their content, but they yielded some different results. Reviews on CBT for obsessive compulsive disorder and social phobia also showed that predictors of effects can be different for similar interventions (Eskildsen et al., 2010; Keeley et al., 2008). More research on different cognitive behavioral interventions is needed to determine whether moderators of effects on CaF will differ for each cognitive behavioral program, or whether there are also variables that consistently appear as moderators.

## Conclusion

In the current study, we used marginal models to assess whether patient characteristics moderated the effects of two cognitive behavioral interventions on CaF in community-dwelling older people. Living situation, fall history, symptoms of depression, perceived general health, ADL disability, cognitive status, and consequences of falling (loss of independence subscale) moderated effects of AMB-NL or AMB-Home. These participant characteristics may be considered in the recruitment of participants and may guide further intervention optimization, although results should be interpreted with caution. This was an exploratory study and more research is needed to confirm the results.

## Supplemental Material

Supplemental Material - Participant Characteristics as Moderators of the Effects of Cognitive Behavioral Interventions on Concerns About Falling: Secondary Analyses of Two Randomized Controlled Trials Click here for additional data file. Supplemental Material for Participant Characteristics as Moderators of the Effects of Cognitive Behavioral Interventions on Concerns About Falling: Secondary Analyses of Two Randomized Controlled Trials by Marlot Kruisbrink, Rixt Zijlstra, Rik Crutzen, Tanja A. C. Dorresteijn, Bjorn Winkens, and Gertrudis I. J. M. Kempen in Journal of Applied Gerontology.
