# Supplementary material for: Participant Characteristics as Moderators of the Effects of Cognitive Behavioral Interventions on Concerns About Falling: Secondary Analyses of Two Randomized Controlled Trials
Source: J Appl Gerontol. 2023 Apr 6;42(8):1877–87. doi: 10.1177/07334648231165904 (PMC10394966; doi:10.1177/07334648231165904)
Supplement: Supplemental Material - Participant Characteristics as Moderators of the Effects of Cognitive Behavioral Interventions on Concerns About Falling: Secondary Analyses of Two Randomized Controlled Trials [file sj-pdf-1-jag-10.1177_07334648231165904.pdf]

Supplementary material

Table 1. Baseline characteristics of analysed samples for AMB-NL and AMB-Home.

Trial	AMB-NL		AMB-Home	
Characteristic	Intervention group (n=280)	Control group (n= 260)	Intervention group (n= 194)	Control group (n= 195)
Concerns about falling[a], mean (SD)	28.50 (9.55)	29.97 (10.16)	35.70 (10.37)	35.47 (9.40)
Demographic				
Age at baseline, mean (SD)	77.5 (4.6)	78.1 (5.0)	78.4 (5.4)	78.3 (5.3)
Female, n (%)	198 (70.7)	190 (73.1)	132 (68.0)	141 (72.3)
Living alone, n (%)	157 (56.1)	138 (53.1)	110 (56.7)	118 (60.5)
Educational level, n (%)				
Low	185 (66.1)	153 (58.8)	110 (56.7)	100 (51.3)
Middle	60 (21.4)	64 (24.6)	55 (28.4)	72 (36.9)
High	35 (12.5)	42 (16.2)	27 (13.9)	22 (11.3)
Health				
Perceived general health, n (%)				
Good	79 (28.2)	86 (33.1)	-	-
Fair	187 (66.8)	161 (61.9)	166 (85.6)	176 (90.3)
Poor	14 (5.0)	13 (5.0)	28 (14.4)	19 (9.7)
Falls in the past 6 months, n (%)				
Never	123 (43.9)	117 (45.0)	64 (33.0)	81 (41.5)
Once	61 (21.8)	48 (18.5)	54 (27.8)	55 (28.2)
More than once	96 (34.3)	95 (36.5)	74 (38.1)	56 (28.7)
ADL disability (range 11-44), mean (SD)	17.14 (4.44)	17.40 (4.53)	18.47 (4.85)	18.70 (4.94)

≥ 1 chronic medical condition, n (%)	110 (39.3)	113 (43.5)	178 (91.8)	186 (95.4)
Cognitive status (range 0-41), mean (SD)	31.69 (3.63)	32.22 (3.86)	-	-
Impaired vision, n (%)	42 (15.0)	46 (17.7)	18 (9.3)	20 (10.3)
Impaired hearing, n (%)	52 (18.6)	51 (19.6)	8 (4.1)	6 (3.1)
Symptoms of depression (range 0-21), mean (SD)	7.17 (4.35)	6.70 (3.94)	6.24 (3.85)	5.96 (3.63)
Feelings of anxiety (range 0-21), mean (SD)	6.85 (3.97)	7.55 (4.66)	6.61 (4.53)	7.12 (4.30)
Socio-cognitive				
Mastery (range 7-35), mean (SD)	21.45 (4.59)	20.92 (4.65)	21.17 (4.80)	20.93 (4.66)
Social support (range 12-48), mean (SD)	28.70 (6.63)	30.42 (6.68)	29.63 (6.45)	29.09 (6.89)
PCOF (range 4-20), mean (SD)	13.48 (3.06)	13.09 (3.11)	13.06 (2.95)	13.19 (2.95)
Perceived consequences of falling - LFI (range 6-24), mean (SD)	14.62 (3.35)	14.90 (3.41)	15.57 (3.29)	15.60 (3.07)
Perceived consequences of falling - DI (range 6-24), mean (SD)	14.73 (2.95)	14.89 (2.95)	15.49 (3.14)	15.88 (2.84)

SD = standard deviation. ADL = activities of daily living. PCOF = perceived control over falling. LFI = loss of functional independence. DI = damage to identity.

Most favorable scores are underlined.

[a]For AMB-NL, concerns about falling were measured with the adapted Falls Efficacy Scale (sumscore range 14 – 56). For AMB-Home, the Falls Efficacy Scale – International was used (sumscore range 16-64).

List of variables in multiple moderator models

AMB-NL, intention to treat	*AMB-NL, on treatment*
Group	Group
Time	Time
Community	Community
Baseline CaF	Baseline CaF
Age	Age
Sex	Sex
Fall history	Fall history
Perceived general health	Perceived general health
Educational level	Educational level
Living situation	Living situation
Hearing impairment	ADL disability
Feelings of anxiety	Symptoms of depression
Symptoms of depression	Cognitive status
Cognitive status	Perceived consequences – loss of functional independence

Perceived consequences – damage to identity<br><br>Group * time<br><br>Sex * time<br><br>Hearing impairment * time<br><br>Feelings of anxiety * time<br><br>Symptoms of depression * time<br><br>Cognitive status * time<br><br>Perceived consequences – damage to identity * time<br><br>Group * sex<br><br>Group * hearing impairment<br><br>Group * feelings of anxiety<br><br>Group * symptoms of depression<br><br>Group * cognitive status<br><br>Group * perceived consequences – damage to identity<br><br>Group * time * sex	Perceived consequences – damage to identity<br><br>Group * time<br><br>Sex * time<br><br>ADL disability * time<br><br>Symptoms of depression * time<br><br>Cognitive status * time<br><br>Perceived consequences – loss of functional independence * time<br><br>Perceived consequences – damage to identity * time<br><br>Perceived general health * time<br><br>Group * sex<br><br>Group * adl disability<br><br>Group * symptoms of depression<br><br>Group * cognitive status<br><br>Group * perceived consequences – loss of functional independence<br><br>Group * perceived consequences – damage to identity

Group * time * hearing impairment<br><br>Group * time * feelings of anxiety<br><br>Group * time * symptoms of depression<br><br>Group * time * cognitive status<br><br>Group * time * perceived consequences – damage to identity	Group * perceived general health<br><br>Group * time * sex<br><br>Group * time * adl disability<br><br>Group * time * symptoms of depression<br><br>Group * time * cognitive status<br><br>Group * time * perceived consequences – loss of functional independence<br><br>Group * time * perceived consequences – damage to identity<br><br>Group * time * perceived general health

AMB-Home, intention to treat	*AMB-Home, on treatment*
Group	Group
Time	Time
Community	Community
Baseline CaF	Baseline CaF
Age	Age
Sex	Sex
Fall history	Fall history
Perceived general health	Perceived general health
Educational level	Educational level
Living situation	Living situation
Visual impairment	Visual impairment
Feelings of anxiety	Social support
Group * time	Perceived consequences of falling
Visual impairment * time	Group * time
Feelings of anxiety * time	Fall history * time

Fall history * time<br><br>Perceived general health * time<br><br>Group * visual impairment<br><br>Group * feelings of anxiety<br><br>Group * fall history<br><br>Group * perceived general health<br><br>Group * time * visual impairment<br><br>Group * time * feelings of anxiety<br><br>Group * time * fall history<br><br>Group * time * perceived general health	Perceived general health * time<br><br>Living situation * time<br><br>Visual impairment * time<br><br>Social support * time<br><br>Perceived consequences of falling * time<br><br>Group * fall history<br><br>Group * perceived general health<br><br>Group * living situation<br><br>Group * visual impairment<br><br>Group * social support<br><br>Group * perceived consequences of falling<br><br>Group * time * fall history<br><br>Group * time * perceived general health<br><br>Group * time * living situation<br><br>Group * time * visual impairment<br><br>Group * time * social support

	Group * time * perceived consequences of falling

AMB-NL, on-treatment outcomes

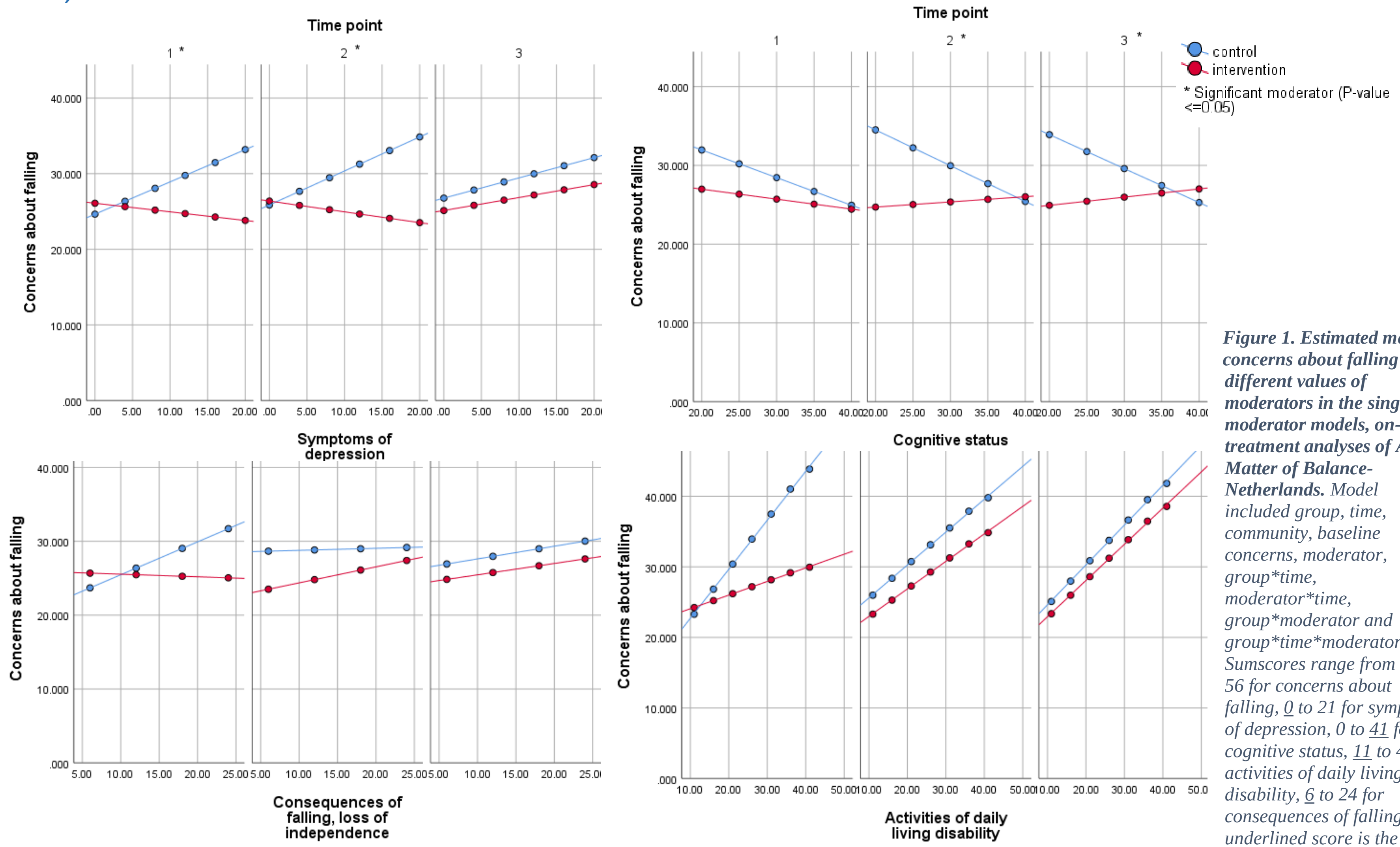

Figure 1. Estimated mean concerns about falling for different values of moderators in the single moderator models, on-treatment analyses of A Matter of Balance-Netherlands. *Model included group, time, community, baseline concerns, moderator, group*time, moderator*time, group*moderator and group*time*moderator. Sumscores range from 14 to 56 for concerns about falling, 0 to 21 for symptoms of depression, 0 to 41 for cognitive status, 11 to 44 for activities of daily living disability, 6 to 24 for consequences of falling (the underlined score is the most favorable score).*

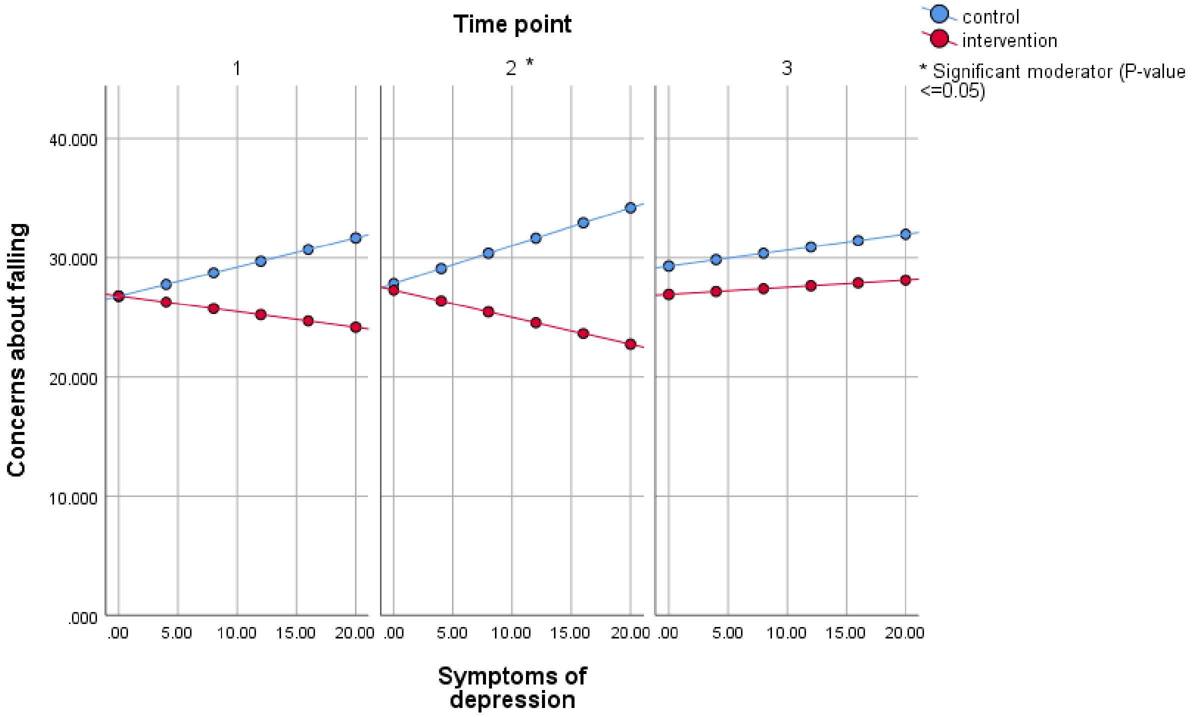

Figure 2. Estimated mean concerns about falling for different values of symptoms of depression in the multiple moderator model, on-treatment analyses of A Matter of Balance-Netherlands. *For adjustments, please see the* ***list of variables in the supplementary material*** *of the article. The Sumscores range from* _14_ *to 56 for concerns about falling and* _0_ *to 21 for symptoms of depression (the underlined score is the most favorable score).*

Table 2. Intervention effects of A Matter of Balance – Netherlands in categories of perceived health in the on-treatment analyses.

Moderator	Time point[a]	Categories[b]	Single moderator model, Adjusted mean difference (95%CI)[c]	**Multiple moderator** model, Adjusted mean difference (95%CI)[d]
Perceived general health	1[e]	Good	1.44 (-1.04; 3.92)	0.15 (-2.49; 2.79)
		Fair	-3.68 (-5.46; -1.91)*	-3.38 (-5.21; -1.56)*
	2	Good	-1.78 (-4.94; 1.38)	-2.64 (-6.02; 0.74)
		Fair	-3.68 (-5.94; -1.42)*	-3.14 (-5.48; -0.79)*
	3	Good	-0.16 (-3.27; 2.96)	-0.83 (-4.21; 2.56)
		Fair	-2.93 (-5.12; -0.74)*	-2.56 (-4.88; -0.24)*

[a]Time point 1 = directly after the intervention, 2 = 6 months after the intervention, 3 = 12 months after the intervention.
[b]Number of people in poor health category was too low to estimate adjusted mean differences; this category is not shown.
[c]Mean difference = Intervention - control. Single moderator model, adjustments for group, time, community, baseline concerns, moderator, group*time, moderator*time, group*moderator and group*time*moderator.
[d]Mean difference = Intervention - control. Multiple moderator model, for adjustments please see the list of variables in the supplementary material of the article.
[e]There is a significant difference (P-value ≤0.05) in intervention effects between categories of the moderator in the single moderator model.

* There is a significant adjusted mean difference between intervention and control group.

AMB-Home, on-treatment outcomes

Table 3. Intervention effects of A Matter of Balance - Home in categories of significant moderators. Results are from the on treatment analysis.

Moderator	Time point [a]	Categories	Single moderator model, Adjusted mean difference (95%CI)[b]	Multiple moderator model, Adjusted mean difference (95%CI)[c]
Living situation	1[d, e]	Alone	-2.33 (-4.50; -0.15)*	0.84 (-2.97; 4.65)
		Not alone	-5.80 (-8.44; -3.16)*	-3.92 (-7.92; 0.09)
	2	Alone	-2.87 (-5.27; -0.48)*	-1.81 (-6.01; 2.40)
		Not alone	-5.29 (-8.23; -2.34)*	-5.30 (-9.80; -0.80)*
Perceived general health	1[d]	Fair	-4.34 (-6.13; -2.55)*	-3.63 (-6.81; -0.45)*
		Poor	1.08 (-3.83; 5.99)	0.56 (-4.78; 5.89)
	2	Fair	-4.42 (-6.40; -2.44)*	-5.89 (-9.51; -2.27)*
		Poor	-0.11 (-5.50; 5.29)	-1.22 (-7.09; 4.66)
Fall in the past 6 months	1	Never	-4.90 (-7.66; -2.13)*	-3.39 (-7.69; 0.90)
		Once	-1.61 (-4.73; 1.50)	-0.02 (-4.33; 4.29)
		More than once	-3.61 (-6.52; -0.69)*	-1.20 (-5.39; 2.99)
	2[d]	Never	-1.77 (-4.83; 1.29)	-2.59 (-7.39; 2.20)
		Once	-2.09 (-5.53; 1.38)	-1.90 (-6.73; 2.92)
		More than once	-7.27 (-10.50; -4.05)*	-6.17 (-10.79; -1.55)*

[a]Time point 1= directly after the intervention, 2= 7 months after the intervention.
[b]Mean difference = Intervention - control. Single moderator model, adjustments for group, time, community, baseline concerns, moderator, group*time, moderator*time, group*moderator and group*time*moderator.
[c]Mean difference = Intervention - control. Multiple moderator model, for adjustments please see the list of variables in the supplementary material of the article.
[d]There is a significant difference (P-value ≤0.05) in intervention effects between categories of the moderator in single moderator model.
[e]There is a significant difference (P-value ≤0.05) in intervention effects between categories of the moderator in multiple moderator model.
* There is a significant adjusted mean difference between intervention and control group.
